# The space of genotypes is a network of networks: implications for evolutionary and extinction dynamics

**DOI:** 10.1038/s41598-017-14048-x

**Published:** 2017-10-23

**Authors:** Pablo Yubero, Susanna Manrubia, Jacobo Aguirre

**Affiliations:** 1 Centro Nacional de Biotecnología, CSIC, c/Darwin 3, 28049 Madrid, Spain; 2 Grupo Interdisciplinar de Sistemas Complejos (GISC), Madrid, Spain

## Abstract

The forcing that environmental variation exerts on populations causes continuous changes with only two possible evolutionary outcomes: adaptation or extinction. Here we address this topic by studying the transient dynamics of populations on complex fitness landscapes. There are three important features of realistic landscapes of relevance in the evolutionary process: fitness landscapes are rough but correlated, their fitness values depend on the current environment, and many (often most) genotypes do not yield viable phenotypes. We capture these properties by defining time-varying, holey, NK fitness landscapes. We show that the structure of the space of genotypes so generated is that of a network of networks: in a sufficiently holey landscape, populations are temporarily stuck in local networks of genotypes. Sudden jumps to neighbouring networks through narrow adaptive pathways (connector links) are possible, though strong enough local trapping may also cause decays in population growth and eventual extinction. A combination of analytical and numerical techniques to characterize complex networks and population dynamics on such networks permits to derive several quantitative relationships between the topology of the space of genotypes and the fate of evolving populations.

## Introduction

The time and mode of response of natural systems to varying environments is a highly challenging subject. Despite the significant progress made in the last decades, many open relevant questions remain. Whole ecosystems are sometimes found to respond smoothly to weak environmental changes, while in other cases critical transitions between states, occasionally causing the extinction of a large number of species, are observed^1–3^. The phenomenology of those transitions was first characterized in theoretical models, and subsequently observed in natural systems. Prominent examples are the desertification of the Sahara^4^, the loss of transparency in shallow lakes^5^ or the dynamics of woodlands in Tanzania^6^. Also, a class of tipping points, where recovery becomes not viable or economically exorbitant has been characterized in ecosystems^7^. These examples notwithstanding, a thorough study of such situations is notoriously difficult, as they typically involve different time scales, several biological organization levels, a variety of non-linear interactions^8^ and a networked structure which commonly entails a whole new phenomenology.

The study of the evolution and adaptation of heterogeneous populations (take as examples viruses or bacteria) in varying environments has recently profited from the use of tools associated to the analysis of dynamical processes on complex networks^9,10^. While the initial emphasis of the application of network theory to natural systems focused on the properties of single networks, recently the interest has turned to understanding how real networks interact with other networks^11^, giving rise to the concept of network of networks or, in a more general context, of multilayer networks^12,13^. For instance, relevant phenomena such as synchronization^14,15^, cooperation^16–18^, robustness^19,20^, transport^21^ or epidemic spreading^22–24^ behave differently when their dynamics occur on a single network or on a network of networks. Furthermore, the dynamics on such architectures often admit a description in terms of competitive scenarios where each network of the ensemble can be depicted as an independent agent struggling with the rest for a certain resource, such as food, wealth, customers or innovation. In this context, it was recently proved that the outcome of such confrontations and the time needed by the winner to prevail drastically depend not only on the internal structure of the competing networks, but also on the connector links, that is, on the structure of the pathways that connect networks^18,25^.

It was long ago suggested that a space of sequences can be mapped into a graph representation where genotypes are the nodes of the graph and links represent their mutual accessibility through mutations^26^, a structure with important implications in population dynamics^27–29^. Though several models have studied neutral networks (the ensemble of all genotypes yielding the same phenotype) in genotype-phenotype maps and described punctuated dynamics of adaptation at the phenotypic level^27,30,31^, we here take a more generic viewpoint where phenotypes need not be explicitly defined and, instead, a fitness value is associated to each genotype. When a fitness-landscape-like structure is linked to time-varying environments, it has been shown that sudden transitions at the genomic scale are likely a generic property of populations and, in analogy to ecosystems, early warning signals that forecast the proximity of such tipping points can be defined^32^. However, the explanation of why these critical genotype transitions occur is incomplete, likely due, as it has previously happened in other systems, to the complexity of the dynamics and interactions unfolding in the space of genotypes, and to an as yet only partial understanding of the overall topology of the latter.

**Figure 1:**
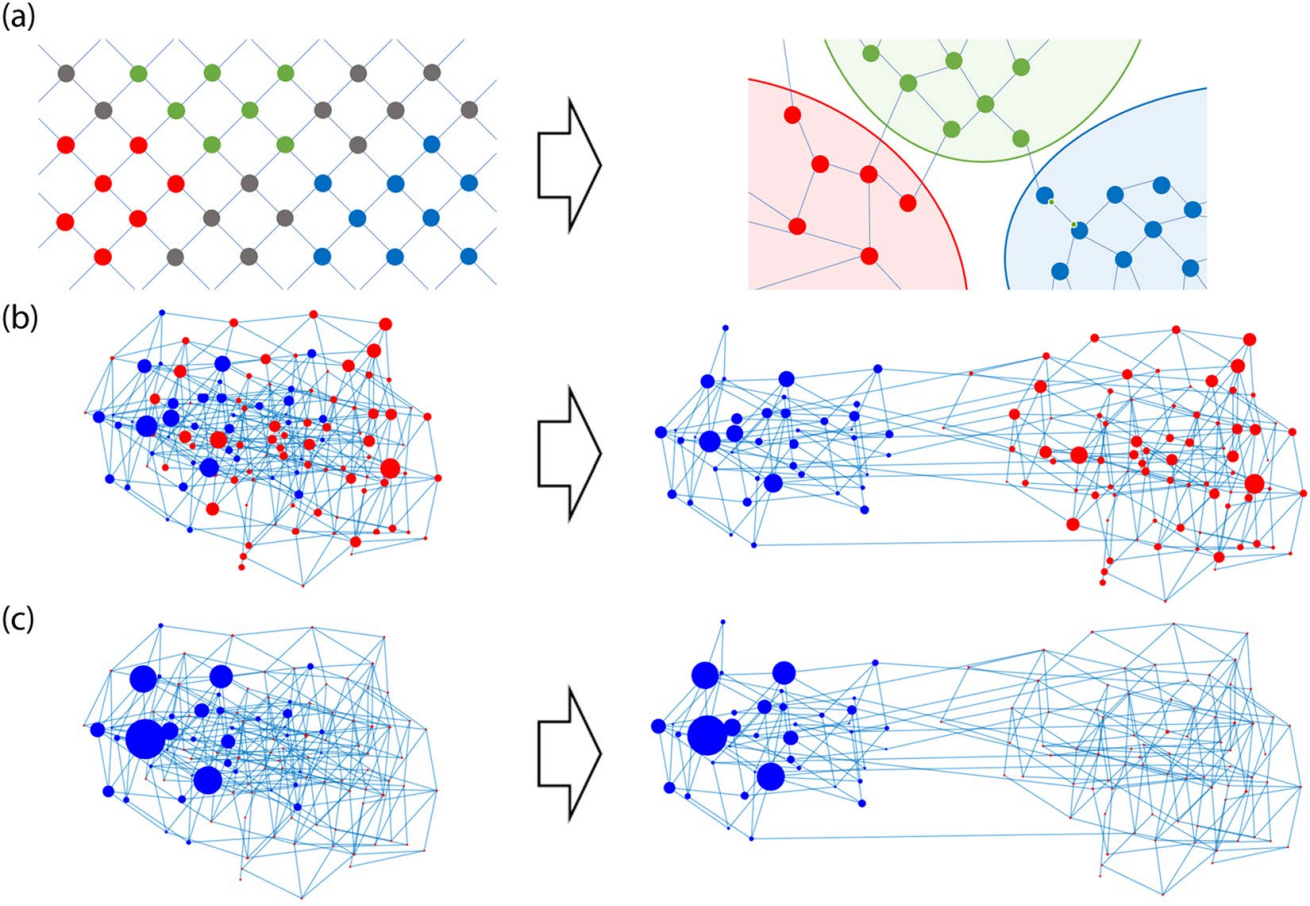
Can the space of genotypes be viewed as a network of networks in competition for population? (**a**) Sketch, low-dimensional representation of a genotype network in which nodes are different genotypes and two nodes are linked if they are mutually accessible through mutations. Nodes in grey have low fitness and are considered non-viable (left), and thus, just attending to connectivity, this part of the network can be analysed as three interconnected networks (right). (**b**,**c**) Genotype space of sequences of length $N=8$ and an alphabet of $A=2$ letters with fitness given by the NK model^33^. Node sizes are proportional to the sequence fitness in (**b**) and to its population at the mutation-selection equilibrium in (**c**). Two communities are identified through Newman’s algorithm^36^. Red and blue colours define the community to which each node belongs to. While in the fitness network (**b**) there is no evidence of the network-of-networks nature of the space of genotypes, the community structure becomes visible when the populations are plotted (**c**).

In this work, and as sketched in Fig. 1, we face two open questions: Can a generic space of genotypes with suitably defined fitness values be described as a network of networks, that is, as a set of networks interconnected through a limited number of connector links? And if this is the case, which are the implications of this structure for population dynamics? In order to address these questions, we first introduce a procedure to construct generic networks mimicking the space of genotypes with an associated fitness landscape. We will use the NK model^33^, which yields landscapes of tunable ruggedness with properties typical of natural landscapes such as epistatic interactions or correlations, multiple peaks, and local optima^34^. A time-varying landscape is generated by suitably interpolating between two NK landscapes. Finally, a variable number of (correlated) genotypes is eliminated so as to recreate the existence of genotypes which do not map onto viable phenotypes. The evolution of populations on such landscapes is studied in various scenarios, in particular for finite and infinite populations affected by different rates of environmental change. We show by a combination of numerical and analytical results that generic genotype spaces do have a structure of a network of networks. A comparison of the results here obtained with the phenomenology derived from competing networks^25,35^ supports that the space of genotypes behaves as a set of communities connected through a limited number of pathways that are crucial for the fate of populations.

## Methods

### The space of genotypes and the NK model

#### Construction of a holey NK fitness landscape

The space of genotypes is formed by all possible sequences of length *N*, with each monomer taken from an alphabet of *A* letters; the resulting network is thus composed of ${A}^{N}$ nodes. This regular network can be represented by its adjacency matrix ${\bf{G}}$, with ${G}_{ij}={G}_{ji}=1$ only if nodes $i$ and $j$ are at a Hamming distance of 1, that is, if sequences $i$ and $j$ differ in only one letter; and ${G}_{ij}=0$ otherwise. Each sequence in the network has exactly $N(A-\mathrm{1)}$ neighbours, since each of its monomers can mutate to $A-1$ letters (see section S1 of the Supplementary Information).

Kauffman’s NK model^33^ is a non-trivial map of genotypes onto fitness, with properties that have been exhaustively analysed in the literature^37–39^. The model has two parameters: $N$ is the length of the sequences (i.e. the number of monomers–nucleotides, aminoacids, etc–per sequence), and $K=\mathrm{0,}\,\mathrm{...,}\,N-1$ is the number of monomers that influence the fitness of any one given (it is analogous to the level of epistasis and determines the degree of correlation among sequences). If $K=0$ the resulting landscape is a smooth *Fujiyama landscape*, with similar genomes having similar fitness values, while when $K=N-1$ the resulting fitness landscape is uncorrelated, i.e. it is a random landscape with the corresponding many local maxima^40^.

The fitness value of the *i*-th sequence is given by

1 $${\tilde{f}}_{i}=\frac{\sum _{j=1}^{N}{s}_{ij}}{N}\,,$$

where each monomer $j$ in a sequence $i$ is assigned a value ${s}_{ij}$ that depends on its $K$ neighbouring monomers. These values ${s}_{ij}$ are taken from a tensor of dimension $N\times {A}^{K+1}$ whose elements are random numbers in the range [0, 1]. A numerical example with detailed computations of fitness values in the NK model is available in section S2 of the Supplementary Information.

It is known that many genotypes are lethal, that is, they do not map to any viable phenotype. Actually, simple models yield examples in a continuum that ranges from the fraction of non-viable genotypes tending to zero with the sequence length, as in RNA, to that same fraction approaching one as genotype size increases^41^. Other models yield intermediate values^42^. The existence of voids of lethal genotypes in genotype space can be effectively captured by introducing the effective fitness of a sequence

2 $${f}_{i}=\{\begin{array}{lll}{\tilde{f}}_{i}-{f}_{l} & {\rm{if}} & {\tilde{f}}_{i} > {f}_{l}\,,\\ 0 & {\rm{if}} & {\tilde{f}}_{i}\le {f}_{l},\end{array}$$

where ${f}_{l}$ is called the *lethality coefficient*. Sequences with ${f}_{i}=0$ represent those genotypes that do not code for viable phenotypes. Moreover, in the NK model sequences of zero effective fitness will tend to form clusters just like in realistic cases^43^ for any $K < N-1$. Throughout this work, the fitness of a sequence is given by Eq. (2).

The fraction of genotypes with fitness equal to zero grows monotonically with ${f}_{l}$, while the number of accessible genotypes with zero fitness (those neighbouring a viable genotype) have a maximum at intermediate values of ${f}_{l}$ (see section S3 of the Supplementary Information).

#### Time-varying fitness landscape

Changes in the environment are frequently driven, showing a value that on average increases or decreases with time^3,44^. We implement this situation by generating an initial $\vec{f}({\tau }_{0})$ and a final $\vec{f}({\tau }_{f})$ fitness landscape as described, where $\tau$ represents the state of the environment, and interpolating linearly between them. The corresponding state of fitness for each genome at environment $\tau$, or equivalently the fitness landscape $\vec{f}(\tau )$ at time $\tau$ is thus defined as

3 $$\vec{f}(\tau )=\vec{f}({\tau }_{0})+\beta (\vec{f}({\tau }_{f})-\vec{f}({\tau }_{0}))\,,$$

where $\beta =\frac{\tau -{\tau }_{0}}{{\tau }_{f}-{\tau }_{0}}\in \mathrm{[0,}\,\mathrm{1]}$ is a parameter that weights the relative contribution of each landscape.

### Model of the evolutionary dynamics of a population

#### Infinite population

Similar to^32^, we consider a population of sequences distributed in the previously defined genotype space. Each sequence produces *r* offspring per generation and has a mutation probability *μ* per genome and replication cycle. The parental population is substituted by its offspring. The transition matrix that represents this evolution with time is

4 $${\bf{M}}=r\mathrm{(1}-\mu ){\bf{F}}+\frac{r\mu }{N(A-\mathrm{1)}}{\bf{FG}}\,,$$

where **G** is the adjacency matrix that encodes the regular topology of the space of genomes, and the diagonal matrix **F** contains the fitness values of each node, ${F}_{ij}={f}_{i}{\delta }_{ij}$, where $i,j=\mathrm{1,}\ldots ,{A}^{N}$. Note that the effective reproduction rate of genome *i* is $r{f}_{i}$ due to the fitness factor.

Each element ${n}_{i}(t)$ of the population vector $\vec{n}(t)$ contains the fraction of population corresponding to the *i*-th sequence at time *t*. The evolutionary process is therefore described by the dynamical equation

5 $$\vec{n}(t+\mathrm{1)}={\bf{M}}\vec{n}(t)\,\mathrm{.}$$

**M** is a primitive matrix^29,45,46^, a property that implies that it has a unique largest eigenvalue ${\lambda }_{1}$ with an associated eigenvector ${\vec{u}}_{1}$ whose components can be chosen so as to have positive entries. Regarding the evolutionary dynamics of the system, these features imply that (i) $\vec{n}(t)$ tends to ${\vec{u}}_{1}$ as $t$ grows, independently of the initial condition $\vec{n}\mathrm{(0)}$ (therefore the population distribution tends to a stable mutation-selection equilibrium at $t\to \infty$ given by ${\vec{u}}_{1}$); (ii) the growth rate of the population at equilibrium (and thus the effective replication rate of all sequences in the population) is ${\lambda }_{1}$; (iii) the time to reach equilibrium verifies, up to first order, ${t}_{eq}\propto ln{({\lambda }_{1}/{\lambda }_{2})}^{-1}$ where ${\lambda }_{2}$ is the second largest eigenvalue of **M** ^29^. We prevent populations from growing indefinitely by normalizing its total size at each iteration $||\vec{n}(t)||=1$, so that in practice we monitor the dynamics of the relative abundance of each sequence.

Finally, note that there are two time scales defined in the model: *t* parametrizes the evolution of a population in a fixed fitness landscape, and $\tau$ is associated to environmental variation. In our simulations for infinite populations we take the limit $t\to \infty$, so that we allow the population to reach equilibrium before the environment changes in a finite amount from $\tau$ to $\tau +1$.

#### Finite population

The evolution with time *t* of a finite population is modelled as follows. The number of offspring of population at node *i* at time *t* is given by $r{f}_{i}(t){n}_{i}(t)$ rounded to the nearest integer. Each new individual has a probability $\mu$ of mutating to one of its $N(A-\mathrm{1)}$ neighbour sequences (both viable and non-viable), and a probability $1-\mu$ of remaining in the same sequence/node as the parental population. A final random Wright-Fisher sampling is applied to maintain the final population within the maximum population limit ${N}_{max}$. Again, the parental population is replaced at each generation.

Since in the case of finite populations we are interested in characterizing the response of the population to environmental variation, and in particular in the likelihood of adaptation versus extinction, we allow for the population to replicate a finite number $t=G\ge 1$ of generations before the next environmental change is applied. *G* depends on each population, so the subjective perception of change is population- or species-dependent. The comparison of these finite time stochastic evolution of populations with the asymptotic, deterministic states obtained with infinite populations permits to quantitatively assess the effects of rapid environmental change and of fluctuations in the population size.

The initial distribution of the population at $\tau =0$ is taken as the mutation-selection equilibrium in the initial environment.

### Analytic solution of evolutionary dynamics in a network of networks

Node centrality stands for the node importance in complex networks theory and can be quantified through different measures. In this work we will use eigenvector centrality, which is given by the entries of the leading eigenvector ${\vec{u}}_{1}$ of the transition matrix **M** ^47^. Note that ${({u}_{1})}_{i} > 0$ for all nodes, and therefore the node centrality is always a positive quantity.

By definition, a network of networks is formed by two or more networks connected through a limited number of connector links. We name connector nodes those nodes of A and B connected through connector links, and **P** is the matrix specifying the latter, that is ${P}_{ij}={P}_{ji}=1$ for links between connector nodes and ${P}_{ij}=0$ otherwise. The first eigenvalue ${\lambda }_{1}$ and eigenvector ${\vec{u}}_{1}$ of the total network formed by networks A and B interconnected, can be expressed as a function of quantities that are only dependent on the properties of isolated networks A and B. Without loss of generality we take ${\lambda }_{1}^{A} > {\lambda }_{1}^{B}$. Developing in powers of the weight $\varepsilon$ of the connector links, the leading terms are

6 $${\vec{u}}_{1}={\vec{u}}_{1}^{A}+\varepsilon \frac{{\vec{u}}_{1}^{A}{\bf{P}}{\vec{u}}_{1}^{B}}{{\lambda }_{1}^{A}-{\lambda }_{1}^{B}}{\vec{u}}_{1}^{B}+{\mathcal{O}}(\varepsilon )\,,$$

7 $${\lambda }_{1}={\lambda }_{1}^{A}+{\varepsilon }^{2}\frac{{({\vec{u}}_{1}^{A}{\bf{P}}{\vec{u}}_{1}^{B})}^{2}}{{\lambda }_{1}^{A}-{\lambda }_{1}^{B}}+{\mathcal{O}}({\varepsilon }^{2})\,,$$

where ${\vec{u}}_{1}^{A}$, ${\lambda }_{1}^{A}$ and ${\vec{u}}_{1}^{B}$, ${\lambda }_{1}^{B}$ are the first eigenvector and first eigenvalue of networks A and B isolated. A full mathematical derivation of those and related quantities can be found in^25,35^.

The centrality of network A (B) is the sum of the centralities of all nodes in network A (B) when $||{\vec{u}}_{1}||=1$. The above equations show that when A is connected to B, centrality becomes redistributed proportionally to the term ${\vec{u}}_{1}^{A}{\bf{P}}{\vec{u}}_{1}^{B}$. This term is a relevant quantity that we call the strength of connections $({S}_{c})$ which can be easily calculated noting that

8 $${\vec{u}}_{1}^{A}{\bf{P}}{\vec{u}}_{1}^{B}=\sum _{cl}{({\vec{u}}_{1}^{A})}_{i}{({\vec{u}}_{1}^{B})}_{j}\,,$$

where $\{cl\}$ is the set of connector links and the sum runs over the products of the eigenvector centralities of connector nodes of A and B, measured when the networks are disconnected.

Finally, it was proved in^25^ that whenever two networks are connected through nodes with little centrality (the so-called peripheral nodes), implying ${\vec{u}}_{1}^{A}{\bf{P}}{\vec{u}}_{1}^{B} \sim 0$, almost all centrality remains in the network with the largest ${\lambda }_{1}$, and even smooth changes in the properties of the networks can yield sharp and drastic centrality redistributions from one network to the other (i.e. genotypic shifts in the context of populations evolving in the space of genotypes, where centrality represents the population at the mutation-selection equilibrium). Furthermore, the time to equilibrium of these dynamical processes significantly increases close to the critical transition. On the contrary, networks connected through connector nodes with large centrality (or central nodes) yield large values of ${\vec{u}}_{1}^{A}{\bf{P}}{\vec{u}}_{1}^{B}$ and spread the centrality over both networks A and B. In this case, smooth changes in the properties of the networks give rise to smooth centrality (or population) redistributions between both networks, and equilibrium is reached fast even in the transition.

### Community detection algorithm

It seems intuitively plausible that a network-of-networks structure can be pinpointed through community detection algorithms^36,48,49^. Most of these algorithms are based on the maximization of the modularity parameter $Q=\frac{1}{4m}{\sum }_{i,j}{B}_{ij}{s}_{i}{s}_{j}$. The modularity matrix **B** has elements ${B}_{ij}={G}_{ij}-\frac{{k}_{i}{k}_{j}}{2m}$, where ${G}_{ij}$ are the entries of the adjacency matrix of the network, ${k}_{i}$ is the number of nodes to which the $i$-th node is linked (its degree), $m$ is the total number of links and ${s}_{i}=1$ or ${s}_{i}=-1$ depending on the community to which node $i$ belongs to. It is straightforward to demonstrate that $Q$ increases with the number of links within communities, and decreases with the number of links between communities (connector links in our case). Indeed, $Q$ is a measure of the goodness of a partition^48^.

The maximization of $Q$ is an NP-hard problem, so approximate methods are commonly used^50,51^. Here we have chosen a method based on the spectral decomposition of the modularity matrix **B** ^36^, originally implemented for non-weighted undirected networks. The method rewrites the modularity parameter as $Q=\frac{1}{4m}{\vec{s}}^{T}{\bf{B}}\vec{s}=$ ${\sum }_{i=1}^{{A}^{N}}{({\vec{v}}_{i}\cdot \vec{s})}^{2}{\beta }_{i}$, such that $Q$ can be computed as a function of **B**’s eigenvalues ${\beta }_{i}$ and its eigenvectors ${\vec{v}}_{i}$. A first approximation to the maximum value of $Q$ is to take the partition vector $\vec{s}$ to be parallel to the eigenvector ${\vec{v}}_{1}$ with largest eigenvalue. However, the entries of $\vec{s}$ can only take the values $\pm 1$, therefore ${s}_{k}=1$ if ${v}_{1k} > 0$ and ${s}_{k}=-1$ if ${v}_{1k} < 0$ where ${v}_{1k}$ is the $k$-th entry of the leading eigenvector ${\vec{v}}_{1}$ of **B**.

Most methods for community detection only focus on the topology of the network, and lack the important biological information related to population dynamics, a quantity affected by topology and by the fitness landscape associated to the network^52^. For this reason, it is convenient to base the partitioning on the topology and on the set of fitness values ${f}_{i}$. For simplicity, we replace the adjacency matrix in the definition of $Q$ by the symmetric weight matrix ${\bf{W}}$, of elements

9 $${W}_{ij}=\sqrt{{G}_{ij}\,{f}_{i}{f}_{j}}\,\mathrm{.}$$

In addition, node degrees ${k}_{i}$ and the total number of links $m$ are replaced by their typical generalization to weighted matrices ${\sum }_{k}{W}_{kj}$ and ${\sum }_{m,n}{W}_{mn}\mathrm{/2}$ respectively^53^. The modularity matrix **B** of a weighted network becomes ${B}_{ij}={W}_{ij}-\frac{({\sum }_{k}{W}_{ik})({\sum }_{l}{W}_{lj})}{{\sum }_{m,n}{W}_{mn}}$.

Note that other definitions of the weight matrix **W** that also take into account both the topology and the fitness landscape could have been used. In particular, the extension to weighted and directed networks of the method for the spectral decomposition of the modularity matrix^53,54^ gives rise to numerical results almost indistinguishable from those obtained with Eq. (9) (see section S4 of the Supplementary Information).

### Definition of relevant quantities

Beyond the above-defined strength of connections ${S}_{c}={\vec{u}}_{1}^{A}{\bf{P}}{\vec{u}}_{1}^{B}$, which is a measure of the underlying network-of-networks structure, we will characterize environmental changes and their effects on populations through three additional quantities.

*Total environmental variability* $(\Delta {f}_{{\tau }_{0}\to {\tau }_{f}})$ quantifies the difference between the initial $\vec{f}({\tau }_{0})$ and final $\vec{f}({\tau }_{f})$ fitness landscapes through

10 $${\rm{\Delta }}{f}_{{\tau }_{0}\to {\tau }_{f}}:=\,1-\frac{\vec{f}({\tau }_{0})\cdot \vec{f}({\tau }_{f})}{||\vec{f}({\tau }_{0}\mathrm{)||\ ||}\vec{f}({\tau }_{f})||}\,,\quad {\rm{\Delta }}{f}_{{\tau }_{0}\vec{}{\tau }_{f}}\in \mathrm{[0,}\,\mathrm{1]}\,\mathrm{.}$$

Values of ${\rm{\Delta }}{f}_{{\tau }_{0}\to {\tau }_{f}}\simeq 0$ mean that the two vectors $\vec{f}({\tau }_{0})$ and $\vec{f}({\tau }_{f})$ are very similar, so a population evolving from one to another is expected to evolve rather smoothly. The opposite occurs with ${\rm{\Delta }}{f}_{{\tau }_{0}\to {\tau }_{f}}\simeq 1$.

In a similar vein we define the $\tau -$ population variability $({\rm{\Delta }}{u}_{{\tau }_{0}\to \tau })$ as the difference between the initial population vector $\vec{u}({\tau }_{0})$ and its value $\vec{u}(\tau )$ at environment $\tau$,

11 $${\rm{\Delta }}{u}_{{\tau }_{0}\vec{}\tau }\,:=\,1-\frac{\vec{u}({\tau }_{0})\cdot \vec{u}(\tau )}{||u({\tau }_{0}\mathrm{)||\ ||}u(\tau )||}\,\mathrm{.}$$

*Maximum genotypic response* $({\rm{\Delta }}{u}_{\tau \to \tau +1})$ is the largest difference between two values of ${\rm{\Delta }}{u}_{{\tau }_{0}\to \tau }$ in consecutive landscapes,

12 $${\rm{\Delta }}{u}_{\tau \to \tau +1}\,:=\,{\rm{\max }}(|{\rm{\Delta }}{u}_{{\tau }_{0}\to \tau +1}-{\rm{\Delta }}{u}_{{\tau }_{0}\to \tau }|)\,,\quad {\rm{\Delta }}{u}_{\tau \to \tau +1}\in \mathrm{[0,}\,\mathrm{1]}\,\mathrm{.}$$

This quantity measures whether the population changes smoothly ${\rm{\Delta }}{u}_{\tau \to \tau +1}\simeq 0$ or abruptly ${\rm{\Delta }}{u}_{\tau \to \tau +1}\simeq 1$ (i.e. it suffers a genotypic shift) under an environmental change of size ${\rm{\Delta }}\tau =1$.

*Average minimum fraction of population* $(\langle {\rho }_{m}\rangle )$ is the normalized average over realizations of the minimum population size ${N}_{min}(t,\tau )$, at any generation $t$ and any environment $\tau$, attained by a finite population under the process of environmental change from $\vec{f}({\tau }_{0})$ to $\vec{f}({\tau }_{f})$,

13 $$\langle {\rho }_{m}\rangle \,:={N}_{max}^{-1}\langle {N}_{min}(t,\tau )\rangle \,,\quad \langle {\rho }_{m}\rangle \in \mathrm{[0,}\,\mathrm{1]}\,,$$

where ${N}_{max}$ is the maximum population size in the simulations. This quantity measures how environmental changes affect the replicative ability of populations; $\langle {\rho }_{m}\rangle$ coincides with the probability of extinction or survival when it takes the limit values 0 or 1, respectively, and for intermediate values is a measure of the relevance of stochastic effects.

## Results

In all simulations the environment linearly changes through 100 different fitness landscapes from the initial $\vec{f}({\tau }_{0}=\mathrm{0)}$ to the final $\vec{f}({\tau }_{f}=\mathrm{100)}$. For computational tractability we will deal here with a space of genotypes of length $N=8$, $K=4$ and an alphabet of $A=2$ letters, therefore with genotype spaces of 256 nodes. In a previous publication^32^, we have shown that genotypic shifts are a generic dynamical property for sequences of any length and any alphabet size evolving on variable fitness landscapes.

### The space of genotypes is a network of networks

Our first goal is to assess whether the space of genotypes can be understood as a network of networks. To face this task, we first analyse a representative example of a population evolving in the space of genotypes with a monotonically varying fitness landscape. Second, we extend the study to a large number of numerical cases to characterize and generalize the results obtained.

#### A representative example

**Figure 2:**
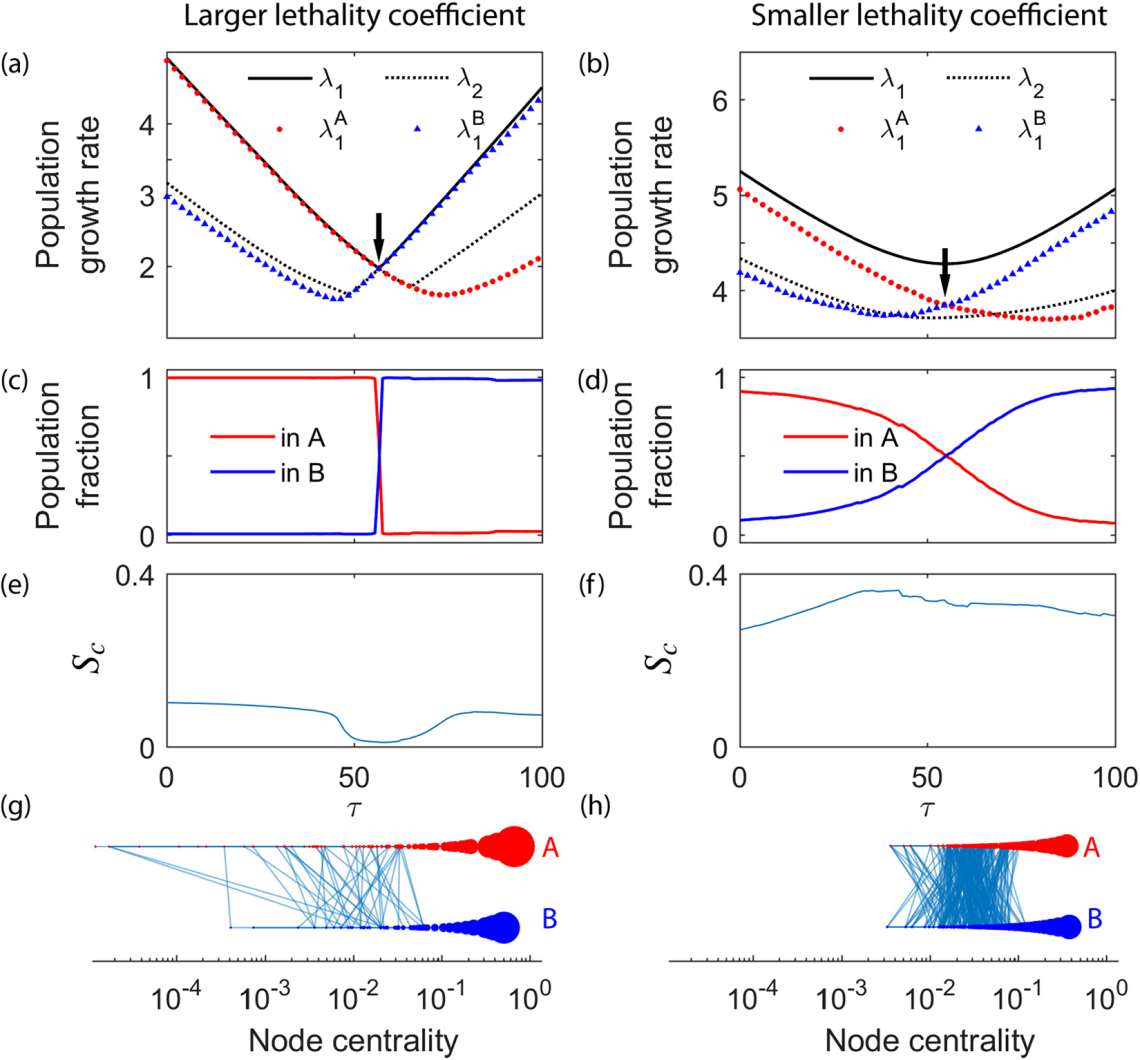
Evolution of an infinite population under environmental changes. Lethality coefficients are ${f}_{l}=0.50$ (**a**,**c**,**e**) and ${f}_{l}=0.30$ (**b**,**d**,**f**). (**a**,**b**) 2 largest eigenvalues ${\lambda }_{1}$ and ${\lambda }_{2}$ of the transition matrix and maximum eigenvalues of isolated communities A and B. (**c**,**d**) Fraction of population within communities A and B. (**e**,**f**) Strength of connections, ${S}_{c}$. (**g**,**h**) Visualization of communities A and B and all connector links for $\tau =55$ (just before the critical value ${\tau }_{c}$ marked with arrows in (**a**,**b**)). The size of each node is proportional to its eigenvector centrality ${({\vec{u}}_{1}^{A})}_{i}$ (respectively B), which is also represented in the $x-$axis.

Figure 2 analyses the evolution of a typical infinite population. As described in Methods, the environment changes from $\tau$ to $\tau +1$ once the population has reached the mutation-selection equilibrium ($t\to \infty$). The results are obtained for a lethality coefficient ${f}_{l}=0.50$ (left column) and ${f}_{l}=0.30$ (right column). These two values cause a significantly heterogeneous and holey landscape or a quite homogeneous network, respectively (specific values of ${f}_{l}$ producing either effect depend on the size of the genotype space). For each value of $\tau$ the whole genotype network is divided in two communities A and B following^36^ (see Methods). Neither community A nor B differed much after successive landscape changes from $\tau$ to $\tau +1$. In fact, over $\mathrm{90 \% }$ of the nodes did not change community in successive steps, supporting the applicability of the community detection algorithm to the case of varying fitness landscapes. In this particular case, we have chosen $\mu =1$ without loss of generality (it has been shown that the magnitude of genotypic shifts increases as the mutation rate decreases, such that high mutation rates are a conservative choice^32^) to emphasize the role played by the second term of Eq. (4), which contains all information on the interaction between topology and fitness in the transition matrix **M**. In fact, mutation rates of the order of one change per genome and replication cycle are frequent^55^, so this situation is coherent with the dynamics of fast-mutating organisms.

In Fig. 2(a,b) we show how the asymptotic growth rate changes in the whole genotype network ${\lambda }_{1}$, in community A $({\lambda }_{1}^{A})$ and in community B $({\lambda }_{1}^{B})$ separately. As it is typical in this kind of systems, a transition takes place when the spectral gap $({\lambda }_{1}-{\lambda }_{2})$ is minimum^29^, a condition that defines the critical value ${\tau }_{c}$. Note that for the larger lethality coefficient ${f}_{l}=0.5$, ${\lambda }_{1}^{A}$ replicates the behaviour of ${\lambda }_{1}$ before the critical transition, and then it is ${\lambda }_{1}^{B}$ which in turn mimics ${\lambda }_{1}$. On the contrary, for ${f}_{l}=0.3$ the asymptotic growth rate of the population in each community does not coincide with that of the whole network, meaning that the latter is relevant for all values of $\tau$.

Figures 2(c,d) show the corresponding evolution of the population: for large values of ${f}_{l}$ a sudden emptying of A at the critical transition time ${\tau }_{c}$ shows the existence of a drastic genotypic shift. This means that before the transition only community A is important, and the rest of nodes do not affect the evolutionary dynamics of the population. The same happens with B after the critical transition. For low values of ${f}_{l}$, however, the transition of the population from community A to community B is smooth.

The results shown in Fig. 2(a–d) support that the dependence of the population distribution and the growth rate on ${f}_{l}$ mimic precisely that of two networks when they are connected through a small number of connector links. That is, the behaviour of the system is precisely that of a network of networks, as described by our Eqs (6–7). The strength of connections appears as the main tuner of this behaviour. Figures 2(e,f) depict the evolution of ${S}_{c}$ for both a large and a low value of ${f}_{l}$ and illustrate their different behaviours. For large ${f}_{l}$, where the genotypic shift is sharp as shown in (c), the strength of connections is very low and in fact deeply falls prior to the transition, while in the second scenario the strength of connections stays practically constant and large, with minor changes all along the process. Finally, in Fig. 2(g,h) we visualize both communities and the connector links just before the transition. In the first case, only a few connector links exist and in fact they mostly connect nodes of low centrality (calculated when communities were isolated, as in Eq. 8), while in the second case many links between large centrality nodes remain.

#### Maximum genotypic response decreases algebraically with the strength of connections

**Figure 3:**
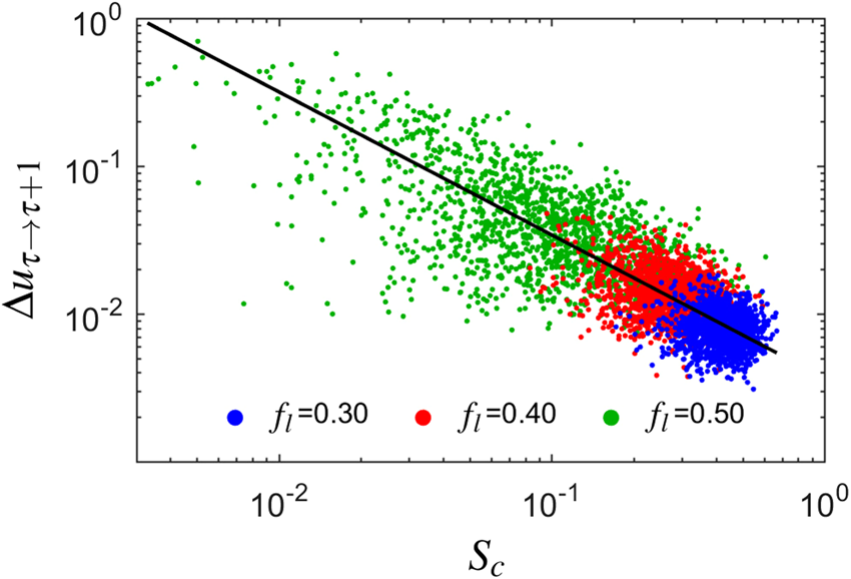
Relation between the maximum genotypic response ${\rm{\Delta }}{u}_{\tau \to \tau +1}$ and the strength of connections ${S}_{c}$ measured at $\tau ={\tau }_{c}$ for different values of the lethality coefficient ${f}_{l}$. The rest of model parameters are as in Fig. 2. We have performed 4500 runs with random initial and final landscapes. The algebraic expression that best fits to the data is plotted in black.

In order to generalize the results obtained in the previous section to a broader range of cases, here we explore the relationship between the maximum genotypic response ${\rm{\Delta }}{u}_{\tau \to \tau +1}$ and the strength of connections ${S}_{c}$ measured at the critical transition time ${\tau }_{c}$ for different values of the lethality coefficient ${f}_{l}$ and different pairs of initial and final landscapes. The results are plotted in Fig. 3.

When ${f}_{l}$ increases, abrupt transitions become common. This is due to an increase in the amount of non-viable genotypes and the concomitant increase in network heterogeneity. Different communities are less connected and through more peripheral nodes. Note, however, that in Fig. 3 smooth shifts might still occur for high values of ${f}_{l}$. The reason is that the randomly chosen initial and final landscapes might be too similar $({\rm{\Delta }}{f}_{{\tau }_{0}\vec{}{\tau }_{f}}\simeq 0)$ so as to permit drastic transitions. The black line depicts the relationship between the maximum genotypic response and the strength of connections. They are related through the algebraic function

14 $${\rm{\Delta }}{u}_{\tau \to \tau +1}\sim a{S}_{c}^{-\alpha }\,,$$

where $a=0.00367$ and $\alpha =0.969$, with a correlation $\rho =-0.821$. To obtain such expression we have taken into account that the joint distribution of two variables that independently follow Gaussian distributions can be approximated by a bivariate normal distribution, where the slope of the major ellipse axis of the scatter plot *a* is obtained from the eigenvectors of the covariance matrix associated to the distribution, and $|\rho |\le 1$ quantifies the degree of correlation between the two variables: the larger $|\rho |$, the narrower the ellipse^56^. We recall that our normalized definition of the maximum genotypic response entails ${\rm{\Delta }}{u}_{\tau \to \tau +1}\le 1$.

### The likelihood of extinction of finite populations increases with the lethality coefficient and with the total environmental variability

With the aim of analysing the implications of a network-of-networks structure on the extinction of populations, we will use in this section a model for finite populations with dynamics as described in Methods. We recall that now the environment changes from fitness landscape $\tau$ to $\tau +1$ after $t=G$ generations of the population which are not sufficient to reach mutation-selection equilibrium, thus mimicking a situation of fast environmental change. In a single stochastic realization extinction occurs when the population decreases down to 0. While extinction is almost certain when the average minimum fraction of population $\langle {\rho }_{m}\rangle \simeq 0$, in the limit of infinite populations extinction occurs when the asymptotic growth rate ${\lambda }_{1} < 1$ for some $\tau \in \mathrm{[0,}\,\mathrm{100]}$.

**Figure 4:**
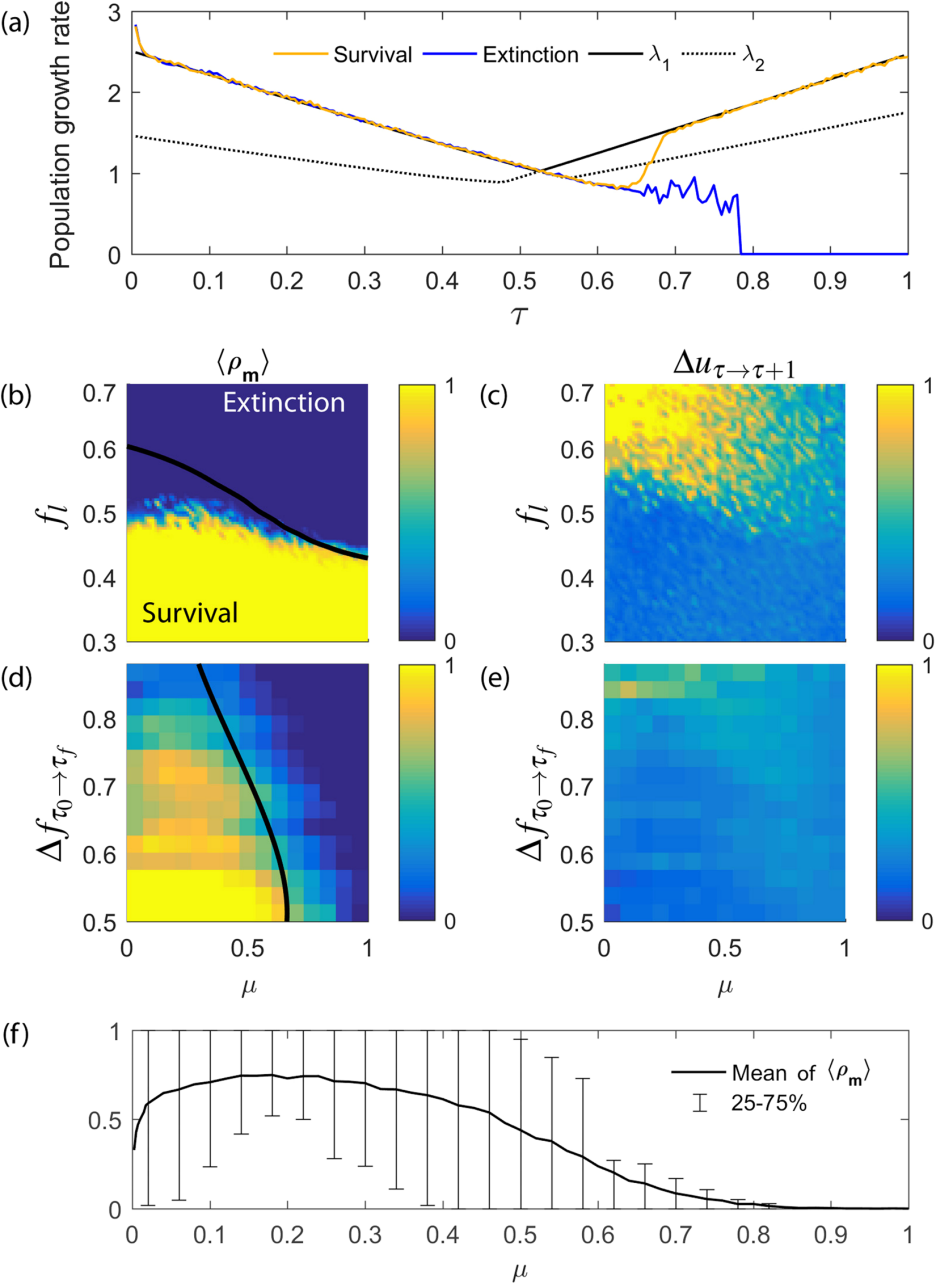
Dependence of the survival of a finite population on the lethality coefficient ${f}_{l}$, mutation rate $\mu$ and total environmental variability ${\rm{\Delta }}{f}_{{\tau }_{0}\to {\tau }_{f}}$. Results have been obtained for a reproduction rate $r=10$, ${N}_{max}={10}^{2}$, $G=1$, and ${f}_{l}=0.5$ when fixed. In section S5 of Supplementary Information different values of ${N}_{max}$ and $G$ are explored. (**a**) Growth rate of two stochastic realizations of the same process with different fates. Eigenvalues ${\lambda }_{1}$ and ${\lambda }_{2}$ of the infinite population model are plotted for comparison. Here $\mu =0.55$. (**b**–**e**) The dependence of the average minimum fraction of population $\langle {\rho }_{m}\rangle$ and the population maximum response ${\rm{\Delta }}{u}_{\tau \to \tau +1}$ (values indicated in colour) on the lethality coefficient ${f}_{l}$ and the mutation rate $\mu$ are plotted in (**b**,**c**) respectively, while their dependence on the total environmental variability ${\rm{\Delta }}{f}_{{\tau }_{0}\to {\tau }_{f}}$ and the mutation rate $\mu$ are plotted in (**d**,**e**). Black thick lines in (**b**) and (**d**) signal the survival-extinction boundary in the infinite-population model, ${\lambda }_{1}=1$. (**f**) Mean of the average minimum fraction of population $\langle {\rho }_{m}\rangle$ over all environments obtained from the data plotted in (**d**). For (**b**–**e**) 12500 simulations were performed (50 values of $\mu \times 250$ values of ${f}_{l}$ or ${\rm{\Delta }}{f}_{{\tau }_{0}\to {\tau }_{f}}$).

Figure 4 summarizes how the survival of a finite population depends on several relevant parameters. We have fixed $G=1$ to study the situation of highest rate of environmental change, where populations will have it difficult to adapt. In Fig. 4(a) the growth rate of two stochastic realizations of the same process, one that eventually survives and another one that goes extinct, are plotted and compared to the corresponding asymptotic growth rate of an infinite population. Note that ${\lambda }_{1} > 1$ for all $\tau$, meaning that an infinite population would always survive for this set of parameters. It is remarkable how the network-of-networks nature of the space of genotypes affects the fate of populations. A finite population gets stuck to the original community A beyond the critical transition (the growth rate now follows ${\lambda }_{2}$, which in fact is the largest eigenvalue of the community A), and can be pushed towards extinction even when there is a different, mutationally accessible community with larger growth rate that would permit its survival. Now adaptation is a stochastic process, and only if an individual mutates so as to traverse a connector link and to find the new community, will the population be able to move to it and avoid extinction (and the growth rate of the population will shift to ${\lambda }_{1}$ again, which now is the maximum eigenvalue of community B).

The dependence of the average minimum fraction of population $\langle {\rho }_{m}\rangle$ and the maximum genotypic response $\Delta {u}_{\tau \to \tau +1}$ on the lethality coefficient ${f}_{l}$, the total environmental variability $\Delta {f}_{{\tau }_{0}\to {\tau }_{f}}$ and the mutation rate $\mu$ are plotted in Fig. 4(b–e) for over ${10}^{4}$ different pairs of initial and final fitness landscapes. As a reference, the survival-extinction boundary for the infinite-population model is plotted in (b) and (d). Those results again emphasize the implications of the network-of-networks structure. First, and extending the results in Fig. 3, the more lethal mutants a population faces, the less strong are the connections joining the two communities. Figures 4(b,c) show that increases in ${f}_{l}$ make transitions from one community to another more abrupt, severely diminishing the chances to survive. Second, if we fix ${f}_{l}$ and increase $\Delta {f}_{{\tau }_{0}\to {\tau }_{f}}$, as shown in (d-e), the different communities will also become more separated in the space of genotypes and as a consequence the survival probability of the population diminishes. Furthermore, $\langle {\rho }_{m}\rangle$ reaches a maximum value for intermediate $\mu$ (Fig. 4(f)). This is also consistent with our network-of-networks perspective. For very low values of $\mu$, the population is gathered around the large fitness genomes of its original community A, the ${S}_{c}$ is low and $\Delta {u}_{\tau \to \tau +1}$ is high. While its average fitness is very large, the population is unable to produce enough genotypic diversity so as to reach and adapt to neighbouring communities. If $\mu$ grows to an intermediate value, the population spreads over a larger region of the genotype space, eventually populating the connector nodes and increasing the strength of connections. In that situation, the survival probability is maximum because, with those border regions of low fitness sufficiently populated, the adaptation to a different community B with larger fitness in case of environmental changes is strongly enhanced. Furthermore, the large value of ${S}_{c}$ will make that transition smoother, and shorter the time to equilibrium. Finally, note that increasing the mutation rate $\mu$ beyond a critical value both in (b) and (d) hinders the population from maintaining its fitter individuals and pushes the whole population to extinction through mutagenic meltdown^57^.

## Discussion

In this work we show that heterogeneous populations evolving on a space of genotypes endowed with a time-varying fitness landscapes can be formally described in the framework of complex network theory, and in particular in terms of competing networks of networks. The equations that describe the competition for resources of evolving populations in the context of networks of networks –our Eqs (6,7)– can be fully applied to this more general biological environment.

In particular, any fitness landscape incorporating correlated roughness, holeyness due to the unavoidable existence of non-viable genotypes, and time variation causes sudden genotypic shifts in species composition and occasionally the total extinction of whole populations. The three conditions above are sufficient, but they are probably not all necessary. For example, we cannot discard that in spaces of very high dimensionality (corresponding to long genotypic sequences) local trapping could arise in absence of holeyness. Shifts and extinction could also occur during the evolution of finite populations on sufficiently large and heterogeneous neutral networks (the ensemble of all genotypes yielding the same phenotype, i.e. in a fixed fitness landscape). Since full genotype spaces for sequences of even moderate length are out of computational reach, network heterogeneity –that is, the existence of a degree distribution different from that of a regular network– appears here mostly linked to the lethality coefficient ${f}_{l}$. This parameter, deeply related to the existence of non-functional genotypes and lethal mutations, tunes the number of connector links and the centrality of the connector nodes. When the number of connector links is low and they connect peripheral nodes, the maximum eigenvalue of the leading community coincides with that of the whole network^25,35^, and the population either remains in the initial or in the final community, with a drastic transition from the former to the latter occurring at a critical value ${\tau }_{c}$, while facing a high extinction probability. On the contrary, when there are many connector links and they connect central nodes, the maximum eigenvalue of the complete network is substantially larger than that of any of its smaller communities, the population is spread over the whole network of genotypes for every environment, the transition between communities is smooth, and the chances are that the population will survive to the environmental change.

Shortened adaptation times caused by faster or more severe environmental changes jeopardize the survivability of populations. High resilience, trapping in suboptimal states and hysteresis under environmental changes have been described in infinite populations^32^. These phenomena are behind the extinction of finite populations, as we have here quantified through different observables. It is important to emphasize that the time to mutation-selection equilibrium, which characterizes the response of populations close to the transition, increases as the distance $|\tau -{\tau }_{c}|$ to the transition decreases. As a consequence, when transitions are sudden this time can be so large that equilibrium cannot be attained whatsoever, even if environmental changes are apparently slow. In natural populations, this means that extinction can occur as the critical threshold is approached, even if the environment is not changing faster than it did at earlier times^32^, and even if the rate of change diminishes but does not halt. An open question of potential relevance for the complex networks community is the nature of this transition in a network-of-networks context^19,58–61^, chiefly if it is continuous or discontinuous in the limit of infinite genotype spaces. In case it is a truly critical transition, it would be important to know about the existence of universal exponents, independent of details of the fitness landscape, characterizing for example the time to equilibrium or the maximum genotypic response.

In the former context, the mutation rate $\mu$ acquires an important role, since the survival probability is maximized at intermediate $\mu -$values. If the mutation rate is too low, an increase in its value makes the transition smoother (and the time to equilibrium concomitantly shorter), but if $\mu$ becomes too large the population might be affected by mutational meltdown^62^. The existence of an optimum value of the mutation rate that maximizes survivability has been often discussed, both in the context of natural systems and in model evolutionary systems, where it becomes a parameter subject to selection^63^. An example are adapting RNA populations, where low values of $\mu$ hinder the capability to efficiently navigate the genotype space, while large values impede the fixation of the solutions eventually reached^64^, and where optimal values of $\mu$ depend on the rate of environmental variation^65^.

Introducing lethal mutations through ${f}_{l}$ as here done induces important correlations between lethal genotypes, in agreement with observations and with the existence of analogous correlations between viable genotypes. Indeed, while a large fraction of genotypes might be non-viable, the fraction of lethal mutations affecting a viable genotype can be much lower (section S3 in Supplementary Information). As a consequence, even in situations where the fraction of viable genotypes vanishes with sequence length (when ${f}_{l}\to 0$ but the dimensionality of the genotype space grows), navigability and a gradual increase in mutational robustness might be preserved^41^.

Several extensions of the scenario here studied support that the phenomenology described does not vary with specifics of the model. The obtained results can be straightforwardly generalized to networks of networks that represent competition among many more than two regions of genotype space^25^ or to include non-linear, partially coupled variations of the fitness of each genotype as time elapses. That situation is the expected one if biological function is depicted as the result of a number of exogenous and endogenous variables that elicit different responses in each genome. Survivability, as measured through the different parameters here introduced, is studied under larger times for adaptation and larger populations in section S5 of the Supplementary Information. As expected, when the maximum population and the interval between environmental changes grow, the results tend to the infinite population limit. A number of extensions that also support the robustness of our qualitative results have been studied previously. They include longer sequences, lower epistasis (higher values of $K$), 4-letter alphabets and lower mutation rates^32^.

Genotype-to-phenotype models have been used to quantify concepts such as navigability of the space of genotypes –for populations evolving on neutral networks– or shape –space covering– a measure of how intermingled different neutral networks are. The latter quantifies the average number of changes a genotype has to experience to reach any of the so-called common phenotypes, and therefore the innovative potential of populations on genotype spaces. Neutral networks with different definitions of fitness have been considered, and important dynamical effects such as certain forms of trapping within phenotypes^66^ and punctuations that alternate with stasis periods have been described: Some models considered an isolated neutral network (thus working in practice with a peak landscape^28,67^), finite populations^68^ and also static fitness landscapes for adapting populations^27^. In one case, the fitness landscape of a pathogenic population varied with the availability of susceptible hosts^31^. The description in terms of network of networks presented in this work is general enough so that it should be applicable to all those scenarios, hopefully providing a common framework where different results can be quantitatively compared.

### Data availability statement

All data generated or analysed during this study are included in this published article (and its Supplementary Information files).

## Electronic supplementary material

Supplementary Information
