# Author Correction: The space of genotypes is a network of networks: implications for evolutionary and extinction dynamics

**DOI:** 10.1038/s41598-018-25218-w

**Published:** 2018-05-03

**Authors:** Pablo Yubero, Susanna Manrubia, Jacobo Aguirre

**Affiliations:** 1 Centro Nacional de Biotecnología, CSIC, c/Darwin 3, 28049 Madrid, Spain; 2 Grupo Interdisciplinar de Sistemas Complejos (GISC), Madrid, Spain

Correction to: *Scientific Reports* 10.1038/s41598-017-14048-x, published online 23 October 2017

This Article contains errors in Reference 32 which is incorrectly given as:

‘Aguirre, J. & Manrubia, S. Tipping points and early warning signals in the genomic composition of populations induced by environmental changes. *Scientific reports* **5** (2015).’

The correct reference is listed below as ref. 1.
