# Supplementary material for: The space of genotypes is a network of networks: implications for evolutionary and extinction dynamics
Source: Sci Rep. 2017 Oct 23;7:13813. doi: 10.1038/s41598-017-14048-x (PMC5653773; doi:10.1038/s41598-017-14048-x)
Supplement: Supplementary file 1 — Supplementary Information [file 41598_2017_14048_MOESM1_ESM.pdf]

Supplementary Information:

The space of genotypes is a network of networks: implications for evolutionary and extinction dynamics,

Pablo Yubero, Susanna Manrubia and Jacobo Aguirre.

Contents

S1 The space of genotypes **2**

S2 The NK model: a numerical example **2**
S2.1 Fitness of AAGG when $K = 0$ . . . 3
S2.2 Fitness of AAGG when $K = 1$ . . . 3

S3 Relation between the topology of the space of genotypes and the lethality coefficient f_l **4**

S4 Robustness of the community detection algorithm **5**

S5 Robustness of the results for finite populations **7**

S1 The space of genotypes

In this section we present a detailed explanation of the network representation of the space of genotypes, that is, the space of all possible sequences –RNA, DNA, viruses...– made of a fixed length N of monomers taken from an alphabet of A letters. Each sequence is a node in the network, and two nodes are linked only if their sequences differ in one letter. Figure S1(a) shows the full network representation of the space of genotypes of length $N = 3$ with monomers taken from an alphabet of $A = 2$ letters: A and G. Every node has exactly $N(A-1) = 3$ neighbours because its $N = 3$ monomers can mutate to $(A-1) = 1$ different letters. As an example, the sequence AAA is highlighted in green and its 3 neighbours in red. Figure S1(b) shows the network representation of a larger space of genotypes, those of length $N = 8$ and $A = 2$. Since all nodes have the same degree, that is, the number of neighbours, the resulting network is regular. Finally, Fig. S1(c) presents a region of a more realistic genotype network. Note that not all genotypes are viable, and thus not all nodes have the same degree. Mutations from the top genotype AAGCACAAGC... are highlighted in red.

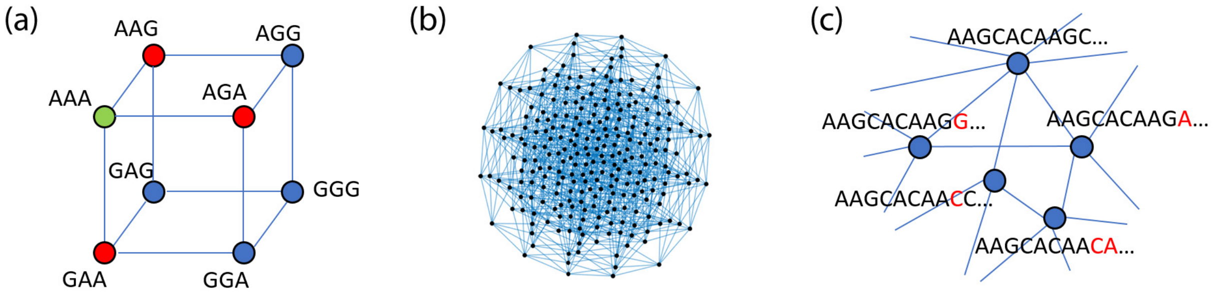

Figure S1. Network representation of the space of genotypes. (a) Space of genotypes of length $N = 3$ and an alphabet of two letters: A and G. Note that the sequence AAA (green node) has three neighbours (red nodes), which differ from it in only one point mutation. (b) Full network representation of a space of genotypes of length $N = 8$ and an alphabet of $A = 2$ letters. (c) Fragment of a realistic genotype network. In red, some mutations from the top sequence are highlighted. In contrast to (a) and (b), not all nodes have the same number of neighbours.

S2 The NK model: a numerical example

In this work we make use of the Kauffman's NK model [1] to map a fitness landscape of tunable ruggedness to the nodes of the network of a space of genotypes (see Methods of the main manuscript). Here we provide for clarity a specific example of its implementation.

First, the model considers a network with sequences of N monomers and a ruggedness level K, where $K \in [0, N-1]$. K tunes the correlation between the fitness of a node and that of its neighbouring nodes.

The fitness of a sequence f_i is the mean value of the fitness of its individual monomers s_{ij},

$$f_i = \frac{\sum_j^N s_{ij}}{N} \,, \tag{S1}$$

where each monomer position j in each sequence i has a value s_{ij} assigned that depends on its K neighbouring monomers. The values s_{ij} are taken from a landscape tensor $\mathfrak{L}$ of dimension $N \times A \times A^K$, which is filled with uniformly distributed random numbers from 0 to 1.

As an example, let us consider a system with a binary alphabet: indices 1 and 2 are assigned to letters A and G respectively, and sequences are of length $N = 4$. In the following subsections, numerical examples for $K = 0$ and $K = 1$ are given for the computation of the fitness of the sequence **AAGG**.

S2.1 Fitness of AAGG when $K = 0$

When $K = 0$, the values s_{ij} for each monomer depend only on the monomer itself. The landscape tensor is therefore of dimension $N \times A$. In this example, we will use the following landscape tensor:

$$\mathfrak{L}_{K=0} = \begin{array}{c} \begin{matrix} j = \text{A} & j = \text{G} \end{matrix} \\ \begin{pmatrix} 0.0974 & 0.9109 \\ 0.7512 & 0.1368 \\ 0.8888 & 0.6625 \\ 0.1508 & 0.1340 \end{pmatrix} \end{array} \begin{matrix} i = 1 \\ i = 2 \\ i = 3 \\ i = 4 \end{matrix}$$

The specific sequence AAGG has in its first and second positions $i = 1, 2$ the letter A, and in $i = 3, 4$ the letter G. Therefore:

$$\begin{aligned} f_{\text{AAGG}} &= \frac{\mathfrak{L}_{1A} + \mathfrak{L}_{2A} + \mathfrak{L}_{3G} + \mathfrak{L}_{4G}}{4} \\ &= \frac{\mathfrak{L}_{11} + \mathfrak{L}_{21} + \mathfrak{L}_{32} + \mathfrak{L}_{42}}{4} \\ &= \frac{0.0974 + 0.7512 + 0.6625 + 0.1340}{4} \\ &= 0.4113\,. \end{aligned}$$

S2.2 Fitness of AAGG when $K = 1$

When $K = 1$ the landscape tensor is of dimension $N \times A \times A$ and the fitness value of the j-th monomer depends on the fitness of its neighbour in other position, for example $j+1$. The tensor can be represented as two matrices of dimension $N \times A$ which correspond (i) to the fitness values when $j =$A, and (ii) when $j =$G. An example of that pair of matrices is given below.

$$\mathfrak{L}_{K=1,j=A} = \begin{array}{c} \begin{matrix} j+1 = \text{A} & j+1 = \text{G} \end{matrix} \\ \begin{pmatrix} 0.9662 & 0.2984 \\ 0.5132 & 0.8885 \\ 0.7129 & 0.3066 \\ 0.5607 & 0.7997 \end{pmatrix} \end{array} \qquad \mathfrak{L}_{K=1,j=G} = \begin{array}{c} \begin{matrix} j+1 = \text{A} & j+1 = \text{G} \end{matrix} \\ \begin{pmatrix} 0.1126 & 0.2270 \\ 0.9355 & 0.2911 \\ 0.0771 & 0.8663 \\ 0.1655 & 0.1352 \end{pmatrix} \end{array}$$

When $K \neq 0$, in Eq. (S1) the term $j = N + 1$ corresponds to $j = 1$ (that is, the sequence is considered cyclic). In fact, as the matrix is filled with uniformly distributed random numbers, the order of interactions does not influence the global properties of the model.

Therefore, the fitness of the sequence AAGG is:

$$\begin{aligned} f_{\text{AAGG}} &= \frac{\mathfrak{L}_{1AA} + \mathfrak{L}_{2AG} + \mathfrak{L}_{3GG} + \mathfrak{L}_{4GA}}{4} \\ &= \frac{0.9662 + 0.8885 + 0.8663 + 0.1655}{4} \\ &= 0.7216\,. \end{aligned}$$

If higher values of K were to be considered, then the dimensionality of the landscape tensor would increase according to $N \times A^{K+1}$ to account for all possible interactions between $K + 1$ monomers.

S3 Relation between the topology of the space of genotypes and the lethality coefficient f_l

Many genotypes are not viable, meaning that they do not map onto a functional phenotype. In consequence, a fraction of all possible genotypes that can be reached through mutations from viable genotypes are lethal. The class of non-viable genotypes is introduced in the model through nodes –i.e. sequences– with fitness below a certain threshold that we call the lethality coefficient f_l: all genotypes with fitness below that threshold are non-viable and their fitness is automatically set to zero (see Eq. (2) of the main manuscript).

The lethality coefficient is an implicit variable which affects the structure of the fitness landscape and the fate of populations. In order to study its biological meaning, we assess here the effects of f_l on two key variables: the fraction of viable nodes (i.e. the fraction of nodes with a fitness greater than f_l), and the fraction of lethal mutations (i.e. the ratio between the number of links connecting a viable to a non-viable node and the number of links that connect one viable node to any other node). This is done for a wide range of fitness landscapes and levels of ruggedness.

Figure S2(a) shows that the fraction of viable genotypes (or nodes, or sequences) decreases monotonically when increasing f_l. It is important to note that there is a value $f_l \sim 0.3$ below which the network does not lose any significant number of nodes, and a value $f_l \sim 0.7$ above which the whole network becomes non-viable. These two phases occur in general, though the specific values depend, among others, on the length of the sequences. Also, the average of the fraction of viable nodes is independent of the ruggedness of the fitness landscape K. In Fig. S2(b) we observe that the fraction of lethal mutations resembles a Gaussian bell centred in $f_l = 0.5$. The decrease when $f_l > 0.5$ is due to the loss of most viable nodes, which are the ones that provide the accessible mutations to the population, and to the unavoidable split into pieces of the network. Moreover, not only the height but also the width of the curve depends on the ruggedness of the fitness landscape: when K grows and in consequence the correlation between different fitness values decreases, non-viable nodes are more and more randomly distributed within the network and thus the maximum number of lethal mutations increases. The opposite also holds: when K decreases, non-viable nodes tend to form clusters thus reducing the number of lethal mutations.

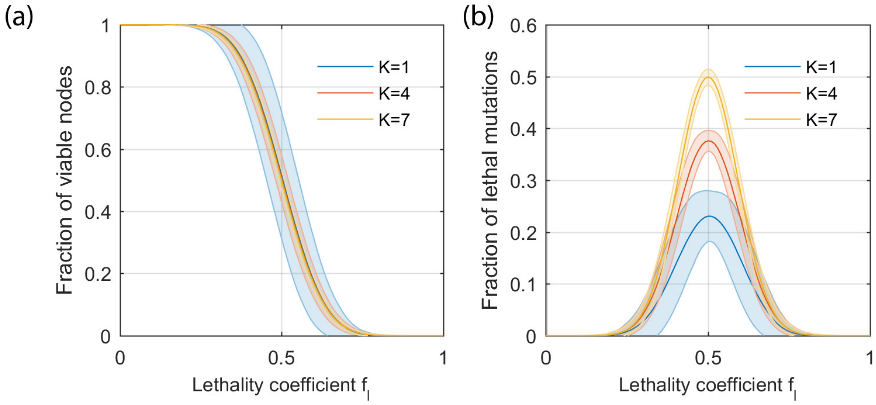

Figure S2. Influence of the lethality coefficient f_l on the space of genotypes. (a) Fraction of viable nodes in a genotype network of length $N = 8$ and $A = 2$ letters as a function of the lethality coefficient for different K. (b) Fraction of lethal mutations as a function of the lethality coefficient for the same three values of K. The shaded areas represent one standard deviation of 500 runs for each K. Observe that the average of the number of viable nodes plotted in (a) does not depend on K, but K strongly influences the fraction of lethal mutations in (b).

S4 Robustness of the community detection algorithm

Choosing the best algorithm to detect communities in a genotype network of networks is still an open question [2]. Accordingly, in this section we compare the results obtained by two different methods, and show that the work presented in this paper is in practice independent of the community detection algorithm implemented.

- **Method 1**: This is the one explained in Methods and implemented throughout the work. It is based on [3, 4]. According to this method, for symmetric weighted networks the elements of the modularity matrix **B** are computed as

$$B_{ij} = W_{ij} - \frac{(\sum_k W_{ik})(\sum_l W_{lj})}{\sum_{m,n} W_{mn}} \,,$$

  and the projection to ± 1 of the components of the leading eigenvector of **B** yields the first approximation to the optimal community division. We have chosen the elements of the weight matrix **W** to be $W_{ij} = \sqrt{G_{ij} f_i f_j}$ (see the main manuscript for more information and the definition of the variables and parameters).

- **Method 2**: We combine the method for weighted networks presented in [3] and the one for directed networks presented in [5]. In this scenario, **B** is computed as in method 1 but with **W**= **M**, where **M** is the transition matrix associated to the dynamical process (Eq. (4) of the main manuscript). In general, **M** and therefore **B** are not symmetric, and thus, instead of computing the leading eigenvector of **B** as in method 1, the community division is given by the leading eigenvector of $\mathbf{B} + \mathbf{B}^T$.

Figure S3 shows the same information as Fig. 2 of the main manuscript, calculated both with method 1 (the one used throughout the paper) and method 2. As it can be clearly seen, the numerical results are hardly distinguishable.

In summary, our results are robust under a change of the algorithm to divide communities, as far as it takes into account the fitness associated to the nodes. The reason is that the boundary nodes that are susceptible to belong to different communities depending on the functional form of W_{ij} will be in general of low centrality and fitness (see Fig. S3(g-h)), and therefore will not affect significantly the population dynamics in either of both communities.

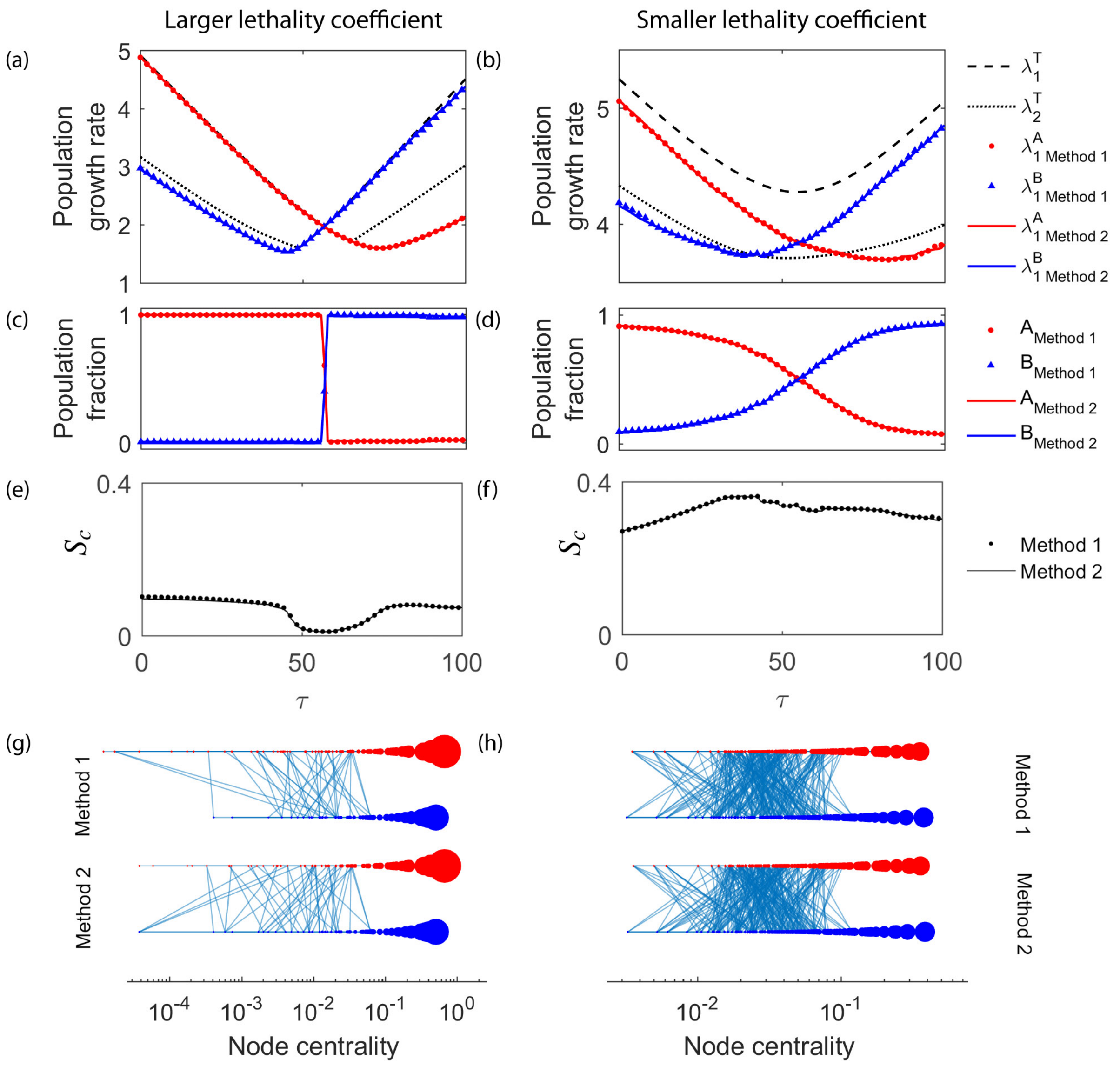

Figure S3. Evolution of an infinite population under environmental changes, making use of 2 different community detection algorithms. Method 1 considers the symmetric weight matrix of elements $W_{ij} = \sqrt{f_i f_j G_{ij}}$ used in Fig. 2 of the main manuscript, whereas method 2 uses the weighted and directed transition matrix $\mathbf{W}$=$\mathbf{M}$. Colours red and blue correspond to communities A and B, and symbols (• and ▲) and solid lines correspond to the community detection methods 1 and 2, respectively. (a,b) 2 largest eigenvalues λ_1 and λ_2 of the transition matrix (dashed and dotted black lines) and maximum eigenvalues of isolated communities. (c,d) Fraction of population within communities A and B. (e,f) Strength of connections, S_c. (g,h) Visualization of communities A and B and all connector links just before the critical value τ_c. The size of each node is proportional to its eigenvector centrality, which is also represented in the $x-$axis. Note in (g,h) that the few nodes that belong to different communities depending on the method used accumulate little centrality.

S5 Robustness of the results for finite populations

In this section we examine the dependence of the extinction dynamics of a finite population in a driven fitness landscape with the maximum population limit N_{max} (maximum number of individuals in the population), and with the number of generations G between consecutive changes in the landscape –which tunes the speed of the environmental change.

An increase in the maximum population limit N_{max} yields a larger number of mutating individuals. The more mutants there are, the higher are the chances of an individual randomly reaching the community with the largest fitness, eventually permitting the survival of the whole population, Fig. S4(b).

For similar reasons, survival is expected to be enhanced as well if the number of generations allowed between consecutive landscape changes increases. In fact, the higher the number of generations G, the easier it is for an individual to find the community with higher fitness, and for the whole population to reach the mutation-selection equilibrium. Results of this scenario are shown in Fig. S4(c).

In summary, increasing the size of a finite population and the time between changes in the environment strongly enhances the survival probability of a genomic population, in agreement with expectations.

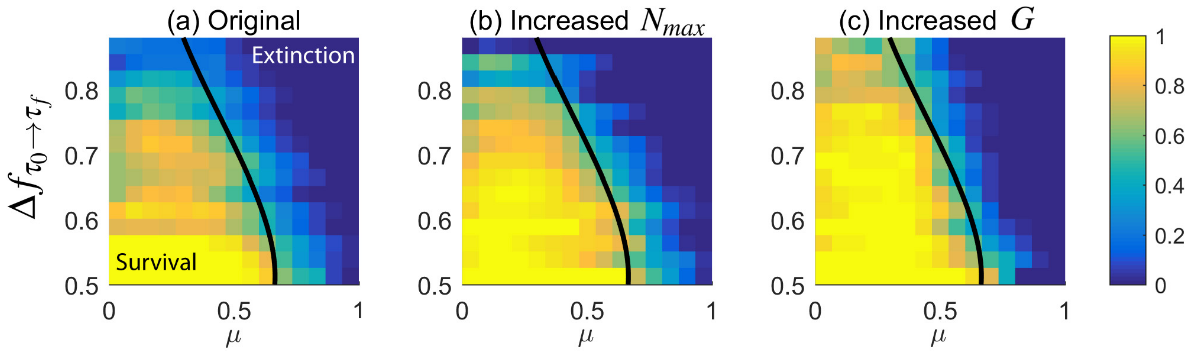

Figure S4. Dependence of the survival of a finite population on the maximum population limit N_{max} and the number of generations G between consecutive environmental changes. The average minimum fraction of population $\langle \rho_m \rangle$ and its dependence on the total environmental variability $\Delta f_{\tau_0 \to \tau_f}$ and the mutation rate μ is shown for three different scenarios. (a) Original parameters $N_{max} = 10^2$ and $G = 1$ as in Fig. 4(b) of the main manuscript. (b) Increased maximum population limit: $N_{max} = 10^4$ and $G = 1$. (c) Increased number of generations G between consecutive landscape changes: $N_{max} = 10^2$ and $G = 10^2$. Data from 10^4 simulations.

References

1. Kauffman, S. A. & Levin, S. Towards a general theory of adaptive walks on rugged landscapes. *Journal of Theoretical Biology* **128**, 11–45 (1987).

2. Capitán J., Aguirre J. & Manrubia S. Dynamical community structure of populations evolving on genotype networks. *Chaos, Solitons & Fractals* **72**, 99–106 (2015).

3. Newman, M. E. J. Analysis of weighted networks. *Phys. Rev. E* **70**, 056131 (2004).

4. Newman, M. E. J. From the Cover: Modularity and community structure in networks. *Proc. Natl. Acad. Sci.* **103**, 8577–8582 (2006).

5. Leicht, E. A. & Newman, M. E. J. Analysis of weighted networks. Community structure in directed networks. *Phys. Rev. Lett.* **100**, 118703 (2008).
